# Spread and impact of the Schmallenberg virus epidemic in France in 2012–2013

**DOI:** 10.1186/s12917-014-0248-x

**Published:** 2014-10-14

**Authors:** Morgane Dominguez, Kristel Gache, Anne Touratier, Jean-Baptiste Perrin, Alexandre Fediaevsky, Eric Collin, Emmanuel Bréard, Corinne Sailleau, Cyril Viarouge, Gina Zanella, Stephan Zientara, Pascal Hendrikx, Didier Calavas

**Affiliations:** Epidemiological Surveillance Unit, French Agency for Food, Environmental and Occupational Health & Safety (ANSES), Maisons-Alfort, France; National animal health farmers’ organisation (GDS France), Paris, France; Animal Health Unit, French General Directorate for Food (DGAL), Ministry of Agriculture, the Food Processing Industry and Forestry, Paris, France; French National Organisation for Veterinary Technical Groups (SNGTV), Paris, France; Maisons-Alfort Laboratory, French Agency for Food, Environmental and Occupational Health & Safety (ANSES), Maisons-Alfort, France; Lyon Laboratory, French Agency for Food, Environmental and Occupational Health & Safety (ANSES), Lyon, France

**Keywords:** Schmallenberg virus, France, Impact, Emerging disease, Sheep, Cattle

## Abstract

**Background:**

The Schmallenberg virus (SBV) emerged in Europe in 2011 and caused a widespread epidemic in ruminants.

In France, SBV emergence was monitored through a national multi-stakeholder surveillance and investigation system. Based on the monitoring data collected from January 2012 to August 2013, we describe the spread of SBV in France during two seasons of dissemination (vector seasons 2011 and 2012) and we provide a large-scale assessment of the impact of this new disease in ruminants.

**Results:**

SBV impact in infected herds was primarily due to the birth of stillborns or deformed foetuses and neonates. Congenital SBV morbidity level was on average moderate, although higher in sheep than in other ruminant species. On average, 8% of lambs, 3% of calves and 2% of kids born in SBV-infected herds showed typical congenital SBV deformities. In addition, in infected herds, farmers reported retrospectively a lower prolificacy during the vector season, suggesting a potential impact of acute SBV infection during mating and early stages of gestation.

**Conclusions:**

Due to the lack of available control and prevention measures, SBV spread quickly in the naive ruminant population. France continues to monitor for SBV, and updated information is made available online on a regular basis [http://www.plateforme-esa.fr/]. Outbreaks of congenital SBV are expected to occur sporadically from now on, but further epidemics may also occur if immunity at population level declines.

## Background

In the autumn of 2011, a previously unknown virus infecting ruminants named "Schmallenberg" virus (SBV) was identified in dairy cows, in the eastern regions of the Netherlands and in north-western Germany [1,2]. This virus belongs to the Simbu serogroup of the genus Orthobunyavirus of the family Orthobunyaviridae. Like other orthobunyaviruses, it is transmitted by arthropod vectors, primarily by biting midges (Culicoides spp). In areas with a temperate climate, this leads to a seasonal pattern of spread (summer and autumn) ([3,4]). Acute SBV infection in adult ruminants has been reported either to cause a mild and transient disease (fever, drop in milk production, diarrhoea in cattle) or to remain clinically unapparent [1,5].

In pregnant ruminants, transplacental SBV infection during a delimited stage of gestation can lead to the birth of severely deformed offspring (i.e. arthrogryposis, stiff neck, brachygnathia, hydranencephaly and other severe brain malformations) ([3,6]). The type of malformation typically caused by viruses of the Simbu serogroup is referred to as “arthrogryposis hydranencephaly syndrome” (AHS) [7,8].

Due to the lack of SBV-specific knowledge, it has been assumed by analogy to Akabane virus (another virus of the Simbu serogroup) that the vulnerable stage of gestation, when SBV foetal infection could lead to AHS, is the second month of gestation for small ruminants (i.e. three to four months before lambing) and between the third and the sixth months of gestation for cattle (i.e. three to seven months before calving) [9-11].

AHS in ruminant offspring due to SBV foetal infections was first reported in Europe during the winter of 2011–2012. As of April 2013, according to the European Food Safety Agency (EFSA), SBV infections had been confirmed in about 9,000 ruminant herds across Europe, and about half of those were reported in France [12].

We present the spatial and temporal patterns of SBV emergence in France. Additionally, we assess the impact of SBV infection within the infected herds.

## Methods

### National framework for emerging animal disease surveillance

Detecting and responding to disease emergence requires robust human and technical capability, capacity and resources. In France, emerging animal disease surveillance and investigation is facilitated through the French platform for epidemiological surveillance in animal health (ESA Platform), which was launched at the end of 2011 to reinforce animal disease monitoring. This platform is coordinated by the French Ministry of Agriculture and involves various animal health stakeholders: national reference laboratories of the French Agency for Food, Environmental and Occupational Health & Safety (ANSES), laboratories (ADILVA, local diagnostic laboratories), livestock organisations (GDS France, Coop de France), a veterinary organisation (SNGTV), an international research centre (Cirad), and wildlife and hunting organisations (FNC and ONCFS).

The ESA Platform is a collaboration framework aiming at improving animal health by supporting the surveillance of established diseases and ensuring the timely detection and effective monitoring and investigation of emerging diseases [13].

### SBV emergence surveillance and investigation system

**Figure 1:**
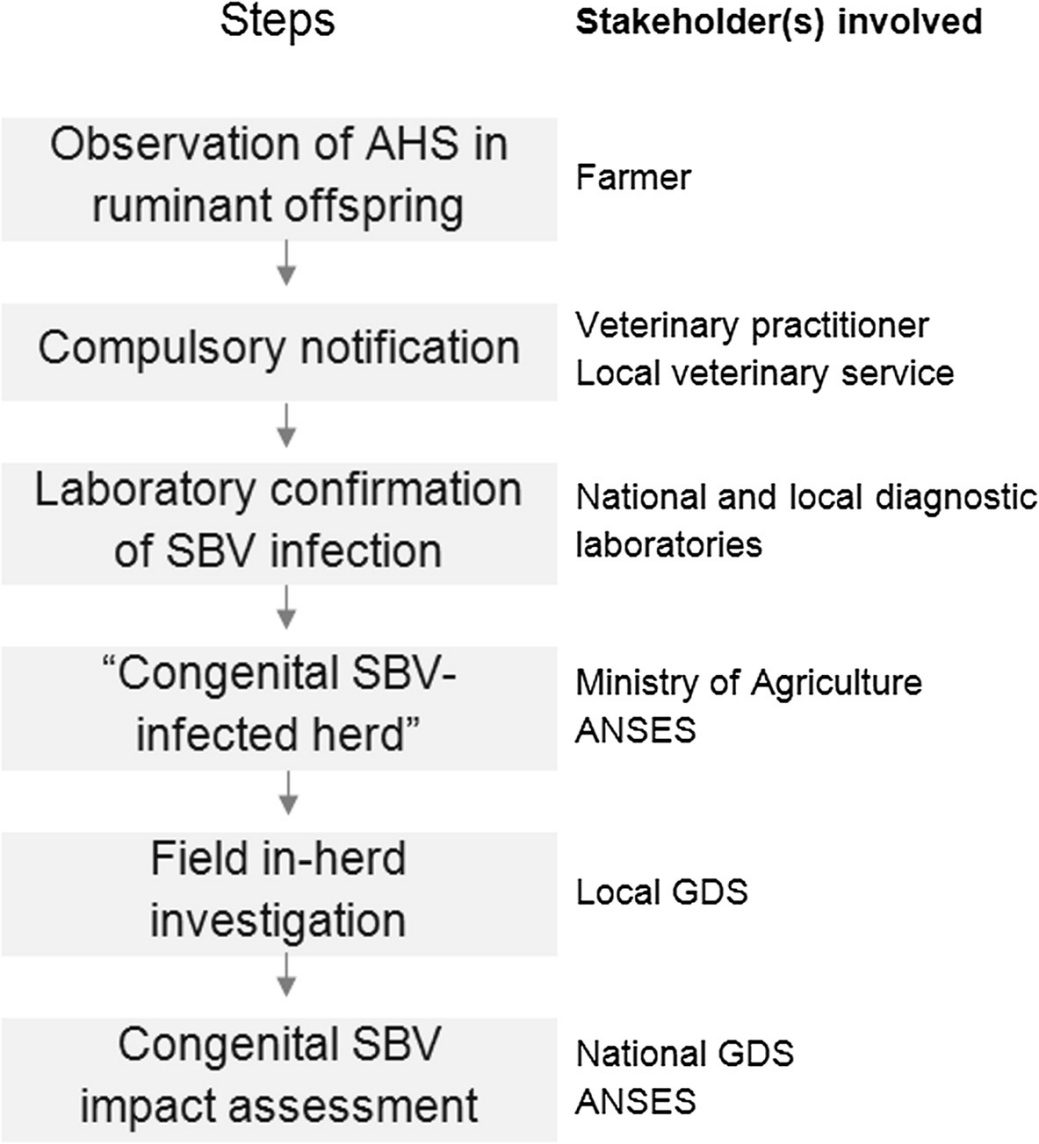
**National multi-stakeholder SBV surveillance and investigation system during the initial stage of alert (France, January – August 2012).**

**Figure 2:**
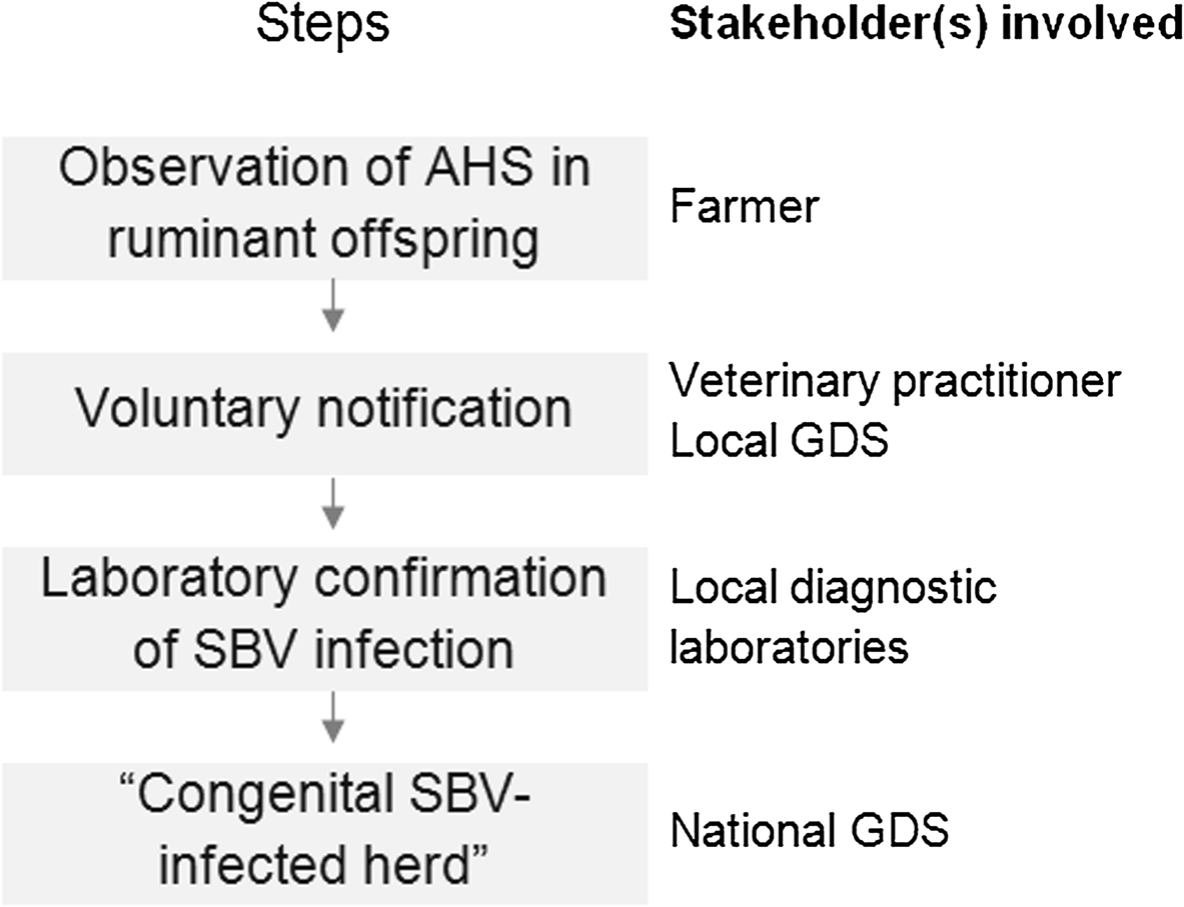
**Simplified national multi-stakeholder SBV surveillance system during the second stage of alert (France, September 2012 – August 2013).**

Since December 2011, following a European alert of the emergence of AHS in ruminants due to SBV infection [14], the ESA Platform has been coordinating the monitoring and investigation of SBV emergence in France. At the onset of the epidemic (from January 2012), the monitoring aimed at assessing the disease situation and providing knowledge on its impact within the infected herds (Figure 1). Then, from September 2012, the level of concern about the SBV disease decreased but the monitoring was continued to keep track of trends in the epidemic (Figure 2).

### Initial stage - maximum level of alert: mandatory reporting of AHS in ruminants and in-herd impact investigations

#### Surveillance of congenital SBV

A “congenital SBV-infected herd” was defined as any ruminant herd with one or more ruminant neonate(s) or foetus(es) showing AHS with a laboratory confirmation of SBV infection.

A national event-based SBV surveillance system based on the mandatory notification of clinical suspicions of AHS in ruminants, coordinated by the Ministry of Agriculture in the framework of the ESA Platform, was launched in early January 2012 to help detect a potential introduction of the virus to France. After the first detection of congenital SBV-infected herds, this surveillance was continued to further assess the spread of the disease [15] (Figure 1).

Farmers were urged to contact their veterinarian when encountering cases of ruminant neonates or foetuses showing AHS. Veterinarians reported suspected cases to the local veterinary services and collected samples for laboratory confirmation of SBV infection. The costs of the veterinarian’s visit and laboratory tests were covered by the government.

SBV diagnosis was performed in national and local reference laboratories. From January to April 2012, SBV infection was confirmed by real-time reverse transcriptase polymerase chain reaction (rtRT-PCR) [1,16], preferentially from offsprings’ brain samples. Upon April 2012, ELISA kits were made available and SBV infection was confirmed by ELISA, preferentially from offsprings’ blood samples collected before colostrum intake [17].

The laboratory results were transmitted to the Ministry of Agriculture, which keeps records at the national level of congenital SBV-infected herds.

#### In-herd impact investigations

Complementary to the surveillance, a national survey was launched to provide an estimate of the impact of the disease in congenital SBV-infected herds (Figure 1). Agents of local animal health farmers' organisations (local GDS) visited the congenital SBV-infected herds identified through the surveillance programme to investigate the impact of the disease according to a standardised investigation questionnaire.

In the herds where births were grouped (e.g. hormonal synchronisation of the oestrous cycle), the impact assessment took into account the births from the beginning of the birth period to the time when the investigation was performed. In herds where the births were not grouped (i.e. some of the beef cattle herds), the impact assessment considered births from three months before the first observation of AHS in that herd to the time when the investigation was performed. The total number of offspring born in congenital SBV affected herds over these periods was recorded by the farmer, as well as the total number of offspring showing various defects (e.g. stillborn or deformed), and among them the number of offspring showing AHS. The types of congenital deformity encountered in the defective offspring were also recorded [18].

At the beginning of the epidemic, any congenital SBV-infected herd identified through surveillance was investigated, provided that the farmer’s agreement was obtained. Then, when more than 30 investigations had been performed for a species in a given département (French administrative unit with a mean area of 5,800 km^2^), only the first in every five congenital SBV-infected herds were investigated. The investigation results were entered by the agents of the local GDS into a national web-based database. The centralised data was analysed jointly by the national animal health farmers’ organisation (GDS-France) and ANSES.

#### Second stage - decreased level of alert: voluntary reporting of AHS in ruminants

In spring 2012, congenital SBV was declared a non-priority disease by both the World Organization for Animal Health [19] and the European Union, so the level of alert for SBV was lowered. Subsequently, the French Ministry of Agriculture disengaged from the coordination of SBV monitoring. From September 2012, in the framework of the ESA Platform, GDS France took over the coordination of a reduced SBV monitoring system aiming at keeping track of trends in the epidemic (Figure 2).

Notifying clinical suspicions of AHS in ruminant neonate(s) or foetus(es) was no longer compulsory, but farmers were strongly encouraged to report any suspicion to their veterinarian. Veterinarians were then asked to report suspected cases to the local GDS and to collect samples for laboratory confirmation. The costs of the veterinarian’s visit and sampling were covered by the government in the framework of the national scheme for abortion surveillance. However, unless specific local agreements had been defined, the laboratory costs for SBV confirmation were incurred by the farmers.

SBV infection was confirmed in local diagnostic laboratories, preferentially by ELISA performed on offsprings’ blood samples collected before colostrum intake. The data collected by the veterinarians in the herds and the SBV laboratory results were entered by GDS agents into a national web-based database. The centralised data was analysed by GDS France.

No specific in-herd impact investigation was performed upon confirmation of infection. However, the data collected by the veterinarians when notifying the suspicion did include a description of the type of congenital deformities observed and also, in order to explore potential consequences of acute SBV infection during the early stages of gestation, a retrospective farmer’s statement on the occurrence of repeated oestrus or early embryonic deaths during the previous vector season.

### Description of the spread and impact of the SBV epidemic in France in 2012–2013

To describe the spread and impact of the SBV epidemic in France in 2012–2013, we analysed the data collected from January 2012 to August 2013 through the two consecutive SBV surveillance and investigation systems. The dataset was based upon data collected from January 2012 to August 2012 through the mandatory reporting of AHS in ruminants and the impact investigation in the affected herds, and the data collected from September 2012 to August 2013 through the voluntary reporting of AHS in ruminants.

Dataset quality was checked for completeness. Duplicates were eliminated using the national herd identification numbers. Only the herds for which a laboratory confirmation for SBV infection was duly recorded were considered as congenital SBV-infected herds.

The dataset was used to compute indicators on the spread of SBV, both temporally (by calculating the number of congenital SBV-infected herds reported by month, from January 2012 to August 2013 – using the date of notification of the clinical suspicion of congenital SBV by the veterinarian), and spatially (by calculating the number of congenital SBV-infected herds reported by Region – i.e. French administrative units corresponding to level 2 of the European nomenclature of territorial units for statistics: “NUTS 2”).

The dataset was also used to perform a univariate descriptive analysis of in-herd impact of congenital SBV, by calculating the median and average frequency of defective offspring.

## Results

### Disease situation

#### Number of reported congenital SBV-infected herds

From January 2012 to August 2013, 4,810 congenital SBV-infected herds were reported in France. Among these, 3,348 (70%) were cattle herds, 1,410 (29%) sheep flocks and 52 (1%) goat flocks (Table 1).

**Table 1:** **Number of congenital SBV-infected herds reported by species (France, January 2012 – August 2013)**

**Herd species**	**Number of herds** ^**1,2**^	**Number of congenital SBV-infected herds**
Cattle	219,000	3,348
Sheep	23,000	1,410
Goat	2,000	52
Total	244,000	4,810

^1^In France (mainland and Corsica).

^2^With cows older than 24 months - ewes older than 12 months - goats older than 12 months.

Overall, by August 2013, congenital SBV infection was reported in 1.5% of the 219,000 cattle farms in France with cows older than 24 months, 6% of the 23,000 sheep farms in France with ewes older than 12 months, and 1% of the 2,000 goat farms in France with goats older than 12 months.

#### Temporal patterns of spread

The first cases of congenital SBV were confirmed in north-eastern France in late January 2012 in lambs that were born in early January. The first cases of congenital SBV reported in goat kids were born in mid-January and the first cases reported in calves were born in late January.

**Figure 3:**
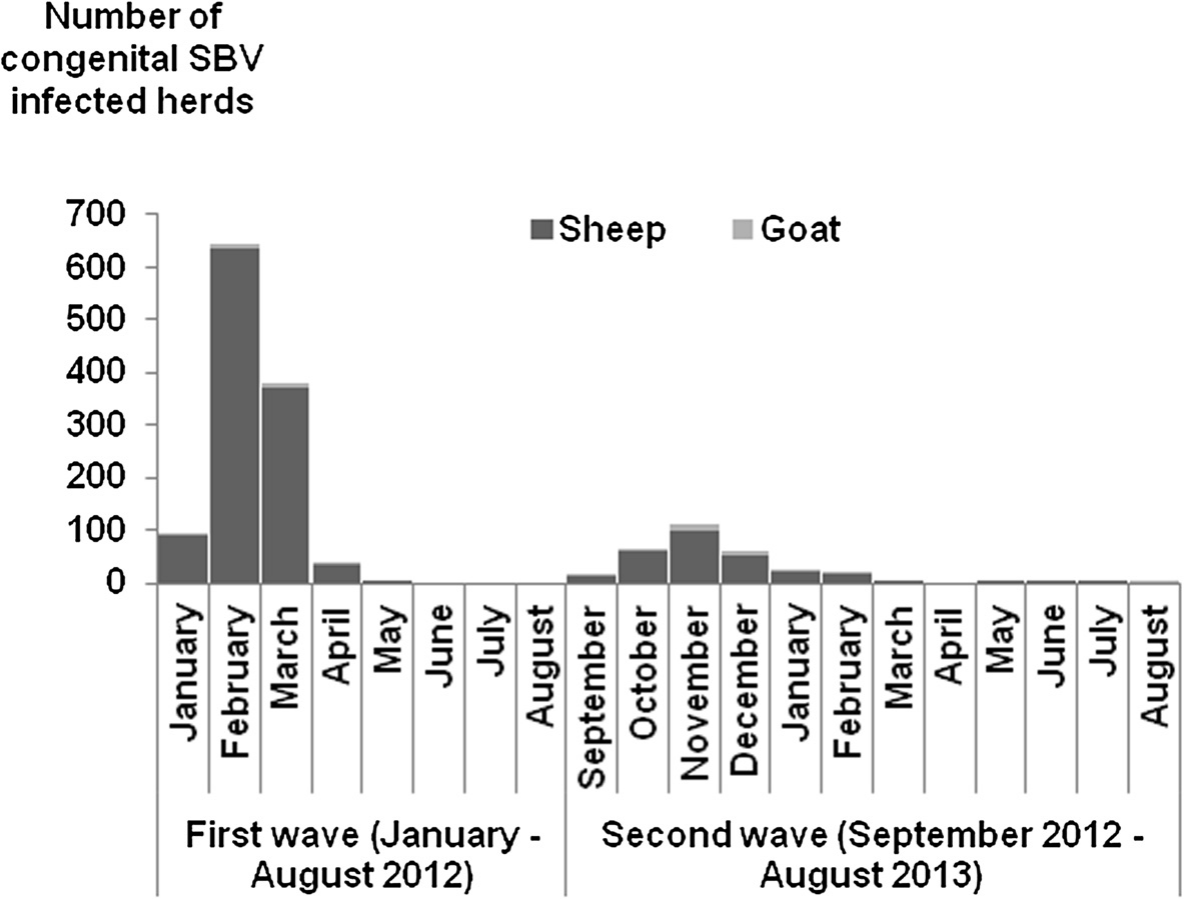
**Number of new small ruminant congenital SBV-infected herds reported per month (France, January 2012 – August 2013).**

**Figure 4:**
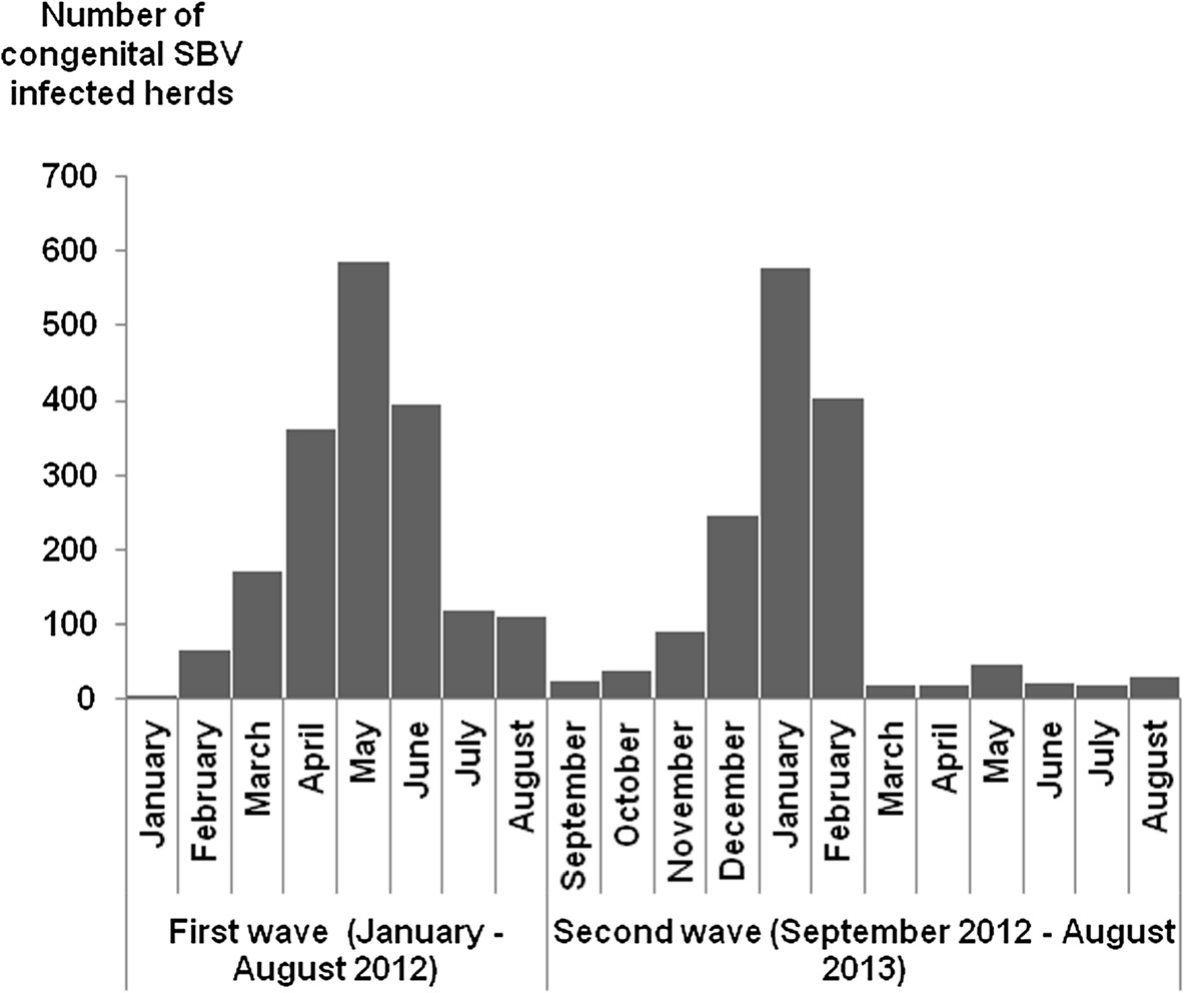
**Number of new cattle congenital SBV-infected herds reported per month (France, January 2012 – August 2013).**

The number of new reports of congenital SBV-infected herds per month evolved over two epidemic waves (Figures 3 and 4). For small ruminants, the first wave of congenital SBV peaked in February 2012 (with over 600 new infected herds reported that month), and then declined rapidly (Figure 3). For cattle, the first wave peaked in May 2012 (with about 600 new infected herds reported that month) and then declined gradually (Figure 4).

The second epidemic wave of congenital SBV began and peaked about four months earlier than the first: the earliest cases were confirmed in cattle and small ruminants in September 2012 (compared to January for the first wave), and the peak was reached in November for small ruminants (compared to February for the first wave), and in January 2013 for cattle (compared to May for the first wave) (Figures 3 and 4).

The two waves of congenital SBV began at the same period for the different ruminant species (i.e. first wave: January 2012; second wave: September 2012). However, the peak occurred about three months later in cattle than in small ruminants (i.e. first wave: May vs February respectively; second wave: January vs November respectively).

#### Spatial patterns of spread

**Figure 5:**
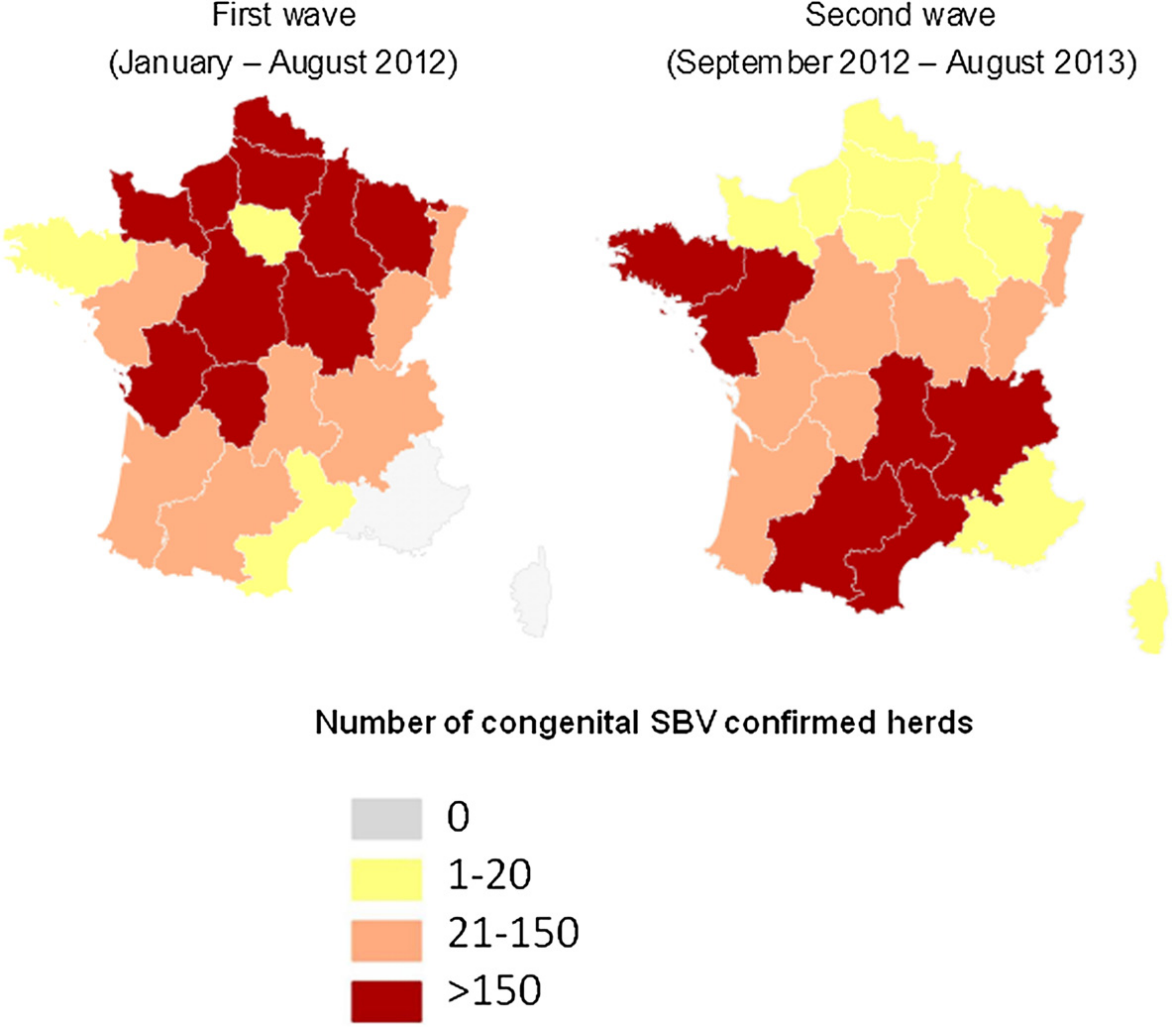
**Number of congenital SBV-infected herds reported by region during the two epidemic waves (France, January 2012 – August 2013).**

**Figure 6:**
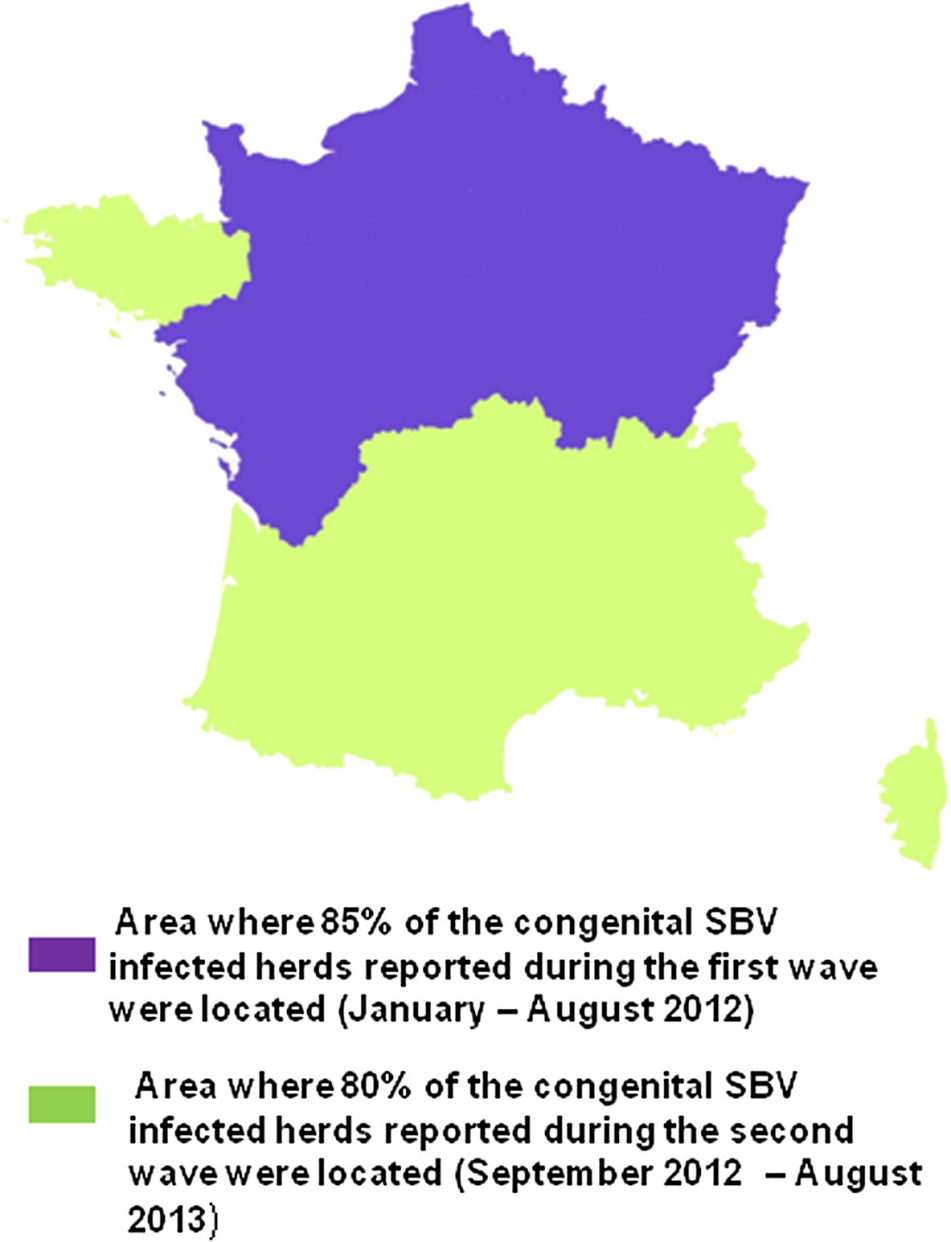
**Areas of concentration of congenital SBV-infected herds reported over the two epidemic waves (France, January 2012 – August 2013).**

SBV spread rapidly over France from the initial north-eastern area of detection. All of the regions but two (Provence-Alpes-Côte d’Azur in south-eastern France and Corsica) were affected during the first wave (Figure 5). The magnitude of the outbreak resulting from the first wave was however much greater in north-eastern France, where about 85% of the reported congenital SBV-infected herds were located (Figure 6).

During the second wave, SBV spread further, and outbreaks were reported throughout France, including Corsica (Figure 5). The magnitude of the outbreak resulting from the second wave was much greater in western and southern areas, where about 80% of the reported infected herds were located (Figure 6).

### Impact assessment

#### In-herd congenital SBV morbidity rate

In-herd impact investigations were performed in over a thousand congenital SBV-infected herds from January to August 2012: 510 infected cattle herds, 612 infected sheep flocks and 12 infected goat herds were investigated, accounting respectively for 23%, 48% and 60% of the infected herds reported during this period.

The median frequency of offspring defects (stillborn, or severely deformed) in the congenital SBV-infected herds was significantly higher in lambs than in calves (Wilcoxon test; p =10^−16^) or kids (Wilcoxon test; p =10^−16^). On average, 8% of the lambs, 3% of the calves and 2% of the kids that were born in the congenital SBV-infected herds showed typical SBV deformities (Table 2). For a given species, there was a significant variability in the morbidity rate between herds.

**Table 2:** **Congenital SBV morbidity in ruminant offspring in reported infected herds (France, January-August 2012, N = 1,011 herds)**

**Offspring species**	**Offspring born in congenital SBV-infected herds**	**Dead or deformed offspring**				**Offspring showing AHS**			
		**Number (n1)**	**Frequency (n1 over N)**			**Number (n2)**	**Frequency (n2 over N)**		
	**Number (N)**		**Median (%)**	**Mean (%)**	**Std dev**		**Median (%)**	**Mean (%)**	**Std dev**
Calves	16,338	1,157	7.5	7.1	18.4	480	3.3	2.9	13.0
Lambs	118,694	18,655	14.0	15.7	30.4	9,311	6.7	7.8	11.8
Kids	937	45	6.6	4.8	27.5	16	6.3	1.7	2.6

#### Deformities observed in the congenital SBV-infected herds

**Figure 7:**
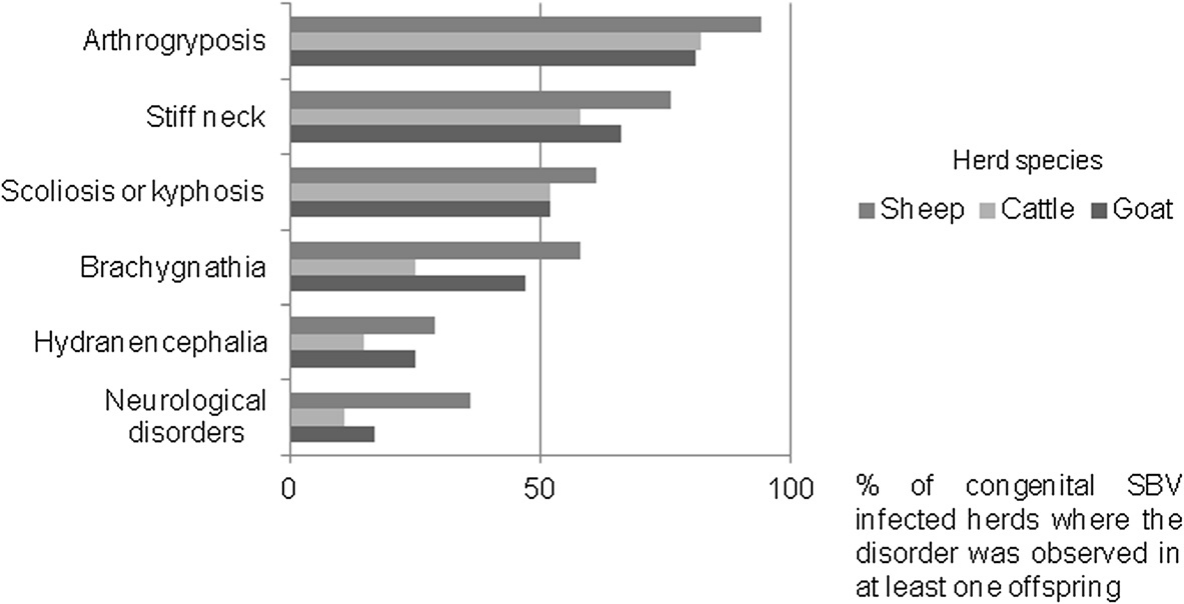
**Average frequency at herd level of disorders encountered in reported congenital SBV-infected herds (France, January 2012 -August 2013, N =1,983 herds).**

The average frequency of deformities was documented in almost 3,000 reported congenital SBV-infected herds from January 2012 to August 2013. For all of the ruminant species, arthrogryposis was the most frequent sign reported in the congenital SBV-infected herds, followed by malformations of the vertebral column (e.g. stiff neck, scoliosis, kyphosis) (Figure 7). Arthrogryposis, which was observed in 94% of the infected sheep flocks, 82% of the infected cattle herds, and 81% of the infected goat flocks, appears to be a sensitive criterion on which to form a clinical suspicion of congenital SBV disease in ruminants.

#### Acute SBV infection & early stages of gestation

Retrospective farmers’ statements on the unusual occurrence of repeated oestrus or early embryonic deaths during the 2012 vector season were obtained in 99% of the congenital SBV-infected herds reported from September 2012 to August 2013. Overall, about 30% of the farmers stated that during the 2012 vector season they had noticed more repeated oestrus or early embryonic deaths than usual (Table 3). This reported frequency was significantly higher in goat and sheep flocks (44% and 35% respectively) than in cattle herds (26%) (Fisher exact test; p = 0.04 and p = 0.02 respectively). In the SBV-infected herds where more frequent repeated oestrus or early embryonic deaths than usual had been noticed during the 2012 vector season, farmers were able to give an estimate of the proportion of females that had been affected: on average, this was 27% of the females in sheep flocks, 15% in cattle herds and 13% in goat flocks.

**Table 3:** **Congenital SBV-infected herds where farmers retrospectively reported more frequent repeated oestrus or early embryonic deaths during the 2012 vector season (France, September 2012 - August 2013, N =1,812 herds)**

**Species**	**Number of congenital SBV-infected herds**	**Number of herds with more frequent repeated oestrus or early embryonic deaths**	
	**N**	**n**	**%**
Cattle	1,509	390	26
Sheep	271	95	35
Goat	32	14	44

## Discussion

Congenital SBV emerged in France during the winter of 2011–2012. A national multi-stakeholder surveillance and investigation system was promptly implemented to monitor the spread of this new disease, and assess its impact in ruminant herds. Field data collected from January 2012 to August 2013 showed that SBV spread rapidly throughout the country and that congenital SBV morbidity levels were generally moderate.

### Spread of SBV epidemic

#### Number of reported outbreaks

The number of outbreaks of congenital SBV reported through surveillance provides important indications about trends in the SBV epidemic but does not provide an accurate estimate of the number of herds that were actually infected by SBV, as some of the SBV-infected herds were either not detected (i.e. infection of non-pregnant ruminants, infection of pregnant ruminants that were not in the susceptible stage of gestation) or not reported. Moreover, under-reporting of congenital SBV may have increased from September 2012 onwards since SBV was no longer a notifiable disease in France, and costs of laboratory analysis for confirmation were sometimes incurred by the farmers.

#### Temporal patterns of spread

Extrapolation of the estimated dates of foetal infection from the dates of birth would indicate that the first reported cases of congenital SBV in France in small ruminants in January 2012 must have resulted from infections that occurred in September or October 2011, while the first reported cases in calves in January 2012 must have resulted from infections from June to October 2011. This suggests that the circulation of SBV in France started in October 2011, at the latest. On the other hand, retrospective serosurveys carried out in two French departments (Meurthe-et-Moselle in north-eastern France where congenital SBV cases were first confirmed in January 2012, and Manche in north-western France where congenital SBV cases were first confirmed in February 2012) showed that SBV was circulating in these departments in October 2011, while the virus was not detected from serum samples collected in August or September 2011 [20]. All together, these data suggest that SBV was not present in France before September 2011.

The seasonality in the number of reported congenital SBV-infected herds is consistent with the seasonality in midge vector activity: each wave of congenital SBV corresponds to cases of congenital SBV resulting from foetal infections that occurred during the previous vector season. The two waves of congenital SBV started approximately at the same period for cattle and small ruminants. This observation is consistent with the minimal interval between foetal infection and birth, which is estimated to be similar for the different ruminant species (three months). It is however noticeable that the epidemic peaked three months later in cattle than in small ruminants, which is due to the longer gestation period and the longer time period during which transplacental infection can lead to foetal damage. The second epidemic wave of congenital SBV began and peaked about four months earlier than the first wave, probably due to an earlier onset of viral circulation in the vector season in 2012 (spring) than in 2011 (autumn).

#### Spatial patterns of spread

The spatial distribution of congenital SBV incidence rates cannot be assessed through surveillance, due on the one hand to the underestimation of the number of infected herds, and on the other hand to the lack of knowledge of the number of susceptible herds (i.e. with pregnant ruminants in the susceptible stage of gestation). However, congenital SBV surveillance data still provide valuable information on the trends of the epidemic’s spread. The findings indicate that the virus spread rapidly across north-eastern France during the first wave. Consistently, serosurveys conducted during the spring of 2012 indicated that SBV within-herd seroprevalence was high in north-eastern France [21]. In that area, outbreaks of congenital SBV were reported rather sporadically during the second wave. Numerous infected herds were identified in southern and western parts of the territory during the second wave. Providing that SBV infection results in long-term protective immunity [12], and despite considerable uncertainty concerning the level of immunity at the population level in the infected areas, the epidemic peak of congenital SBV seems behind us, even if sporadic outbreaks of congenital SBV resulting from the infection of previously unexposed animals are still being reported during the third epidemic wave. As of the 15th of August 2014, 108 congenital SBV-infected herds had been reported throughout France since the first of September 2013 during surveillance of the third wave [22]. Comparatively, 2,976 congenital SBV-infected herd were reported during thefirst wave, and 1,834 during the second wave.

### SBV impact assessment

In the infected herds, SBV impacts were primarily due to the birth of stillborns or deformed foetuses and neonates. While all of the abortions or stillbirths observed in the infected herds might not have been due to SBV infection, congenital deformities known to be fairly specific manifestations of SBV congenital infection represent a valuable indicator of SBV impact (even if the imputability of SBV virus in the occurrence of the disorders or deformities reported has not been ascertained). On average, 8% of the lambs, 3% of the calves and 2% of the kids born in SBV-infected herds showed SBV-typical deformities (AHS)s. This provides a rough estimate of the direct impact of SBV virus infection, although it should be noted that this is only based on farmers’ statements, and a potential information bias cannot be ruled out.

Such a large scale impact study is so far unique in Europe. Our estimate of the median morbidity rate in offspring is consistent with the results of smaller-scale studies carried out in Belgium [23]. For a given ruminant species, there is great variability in the morbidity rate of congenital SBV that could be influenced by within-herd seroprevalence, husbandry and reproduction practices, etc., as shown elsewhere [2].

Although the direct impact of acute SBV infection on adult ruminants has so far been considered as minor, some farmers reported decreases in reproductive capability in the infected herds during the previous vector season, suggesting a potential impact of acute SBV infection on reproduction performance (increased proportion of females returning to oestrus and increased proportion of non-pregnant females), presumably due to foetal death caused by SBV infection of naive females in early stages of gestation. Such observations were also reported by farmers in the Netherlands [24] and Belgium [25]. Specific studies are planned to further explore the impact of SBV infection during the early stages of gestation and to assess whether the reproductive capability is durably damaged.

### Monitoring an emerging disease situation

About half of the SBV outbreaks reported at the European level as of April 2013 were located in France [12]. That does not imply that the disease incidence has been higher in France than in neighbouring countries, but may rather result from the surveillance of the epidemic spread in France, which has greatly benefited from the quality of collaboration across sectors in the framework of the ESA Platform. Indeed, in the context of SBV emergence, the ESA Platform proved to be a valuable tool for enhancing stakeholder coordination, building synergy between disciplines and sectors, and relaying surveillance efforts over time to make it sustainable.

## Conclusion

SBV rapidly spread across France upon its emergence in the autumn of 2011. After two seasons of SBV circulation, all the regions had been infected. France continues to monitor congenital SBV outbreaks, and updated information is made available online on a regular basis [http://www.plateforme-esa.fr/]. Outbreaks of congenital SBV may occur sporadically from now on, unless further epidemics occur if the immunity at population level declines.

### Animal ethics

Our work did not include any experimental research on animals. Data was collected on the farms only if the farmer’s agreement (informed consent) had been obtained.

## References

[1] Hoffmann B, Scheuch M, Höper D, Jungblut R, Holsteg M, Schirrmeier H, Eschbaumer M, Goller KV, Wernike K, Fischer M, Breithaupt A, Mettenleiter TC, Beer M: **Novel orthobunyavirus in cattle, Europe, 2011.** *Emerg Inf Dis* 2012, **18**(2). http://dx.doi.org/10.3201/eid1803.111905. 10.3201/eid1803.111905 PMC3309600 22376991

[2] Veldhuis A, van Schaik G, Vellema P, Elbers A, Bouwstra R, van der Heijden H, Mars M (2013). Schmallenberg virus epidemic in the Netherlands: spatiotemporal introduction in 2011 and seroprevalence in ruminants. Prev Vet Med.

[3] Conraths FJ, Peters M, Beer M: **Schmallenberg virus, a novel orthobunyavirus infection in ruminants in Europe: Potential for global impact and preventive measures.** *New Zeal Vet J* 2013. doi:10.1080/00480169.2012.738403. 10.1080/00480169.2012.738403 23215779

[4] Scholte EJ, Mars MH, Braks M, Den Hartog W, Ibañez-Justicia A, Koopmans M, Koenraadt JCM, De Vries A, Reusken C: **No evidence for the persistence of Schmallenberg virus in overwintering mosquitoes.** *Med Vet Entomol* 2013. Epub. 10.1111/mve.12010 23692132

[5] Wernike S, Hoffmann B, Bréard E, Botnerc A, Ponsart C, Zientara S, Lohsec L, Pozzid N, Viarouge C, Sarradine P, Leroux-Barce C, Rioue M, Laloy E, Breithauptf A, Beer M (2013). Schmallenberg virus experimental infection of sheep. Vet Microbiol.

[6] EFSA (2012). Schmallenberg Virus: Analysis of the Epidemiological Data.

[7] Coverdale O, Cybinski D, St. George T (1978). Congenital abnormalities in calves associated with Akabane virus and Aino virus (Letter). AVJ.

[8] Matumoto M, Inaba Y (1980). Akabane disease and Akabane virus. Kitazato Arch Exp Med.

[9] Hashiguchi Y, Nanba K, Kumagai T (1979). Congenital abnormalities in newborn lambs following Akabane virus infection in pregnant ewes. Natl Inst Anim Health Q.

[10] Kirkland PD, Barry RD, Harper PA, Zelski RZ (1988). The development of Akabane virus-induced congenital abnormalities in cattle. Vet Rec.

[11] Martinelle L, Pozzo Dal F, Kirschvink N, De La Grandière MA, Thiry E, Saegerman C (2012). Le virus Schmallenberg ou l'émergence du premier orthobunyavirus du sérogroupe simbu en Europe. Ann Med Vet.

[12] EFSA (2013). Schmallenberg Virus: Analysis of the Epidemiological Data.

[13] Calavas D, Fediaevsky A, Collin E, Touratier A, Amar P, Mocquay V, Marcé C, Bronner A, Hendrikx P (2012). Plateforme nationale de surveillance épidémiologique en santé animale, missions prioritaires et organisation. Bull Epid Santé Anim Alim.

[14] Van den Brom R, Luttikholt S, Lievaart-Peterson K, Peperkamp N, Mars M, van der Poel W, Vellema P (2012). Epizootic of ovine congenital malformations associated with Schmallenberg virus infection. Tijdschr Diergeneeskd.

[15] Doceul V, Lara E, Sailleau C, Belbis G, Richardson J, Breard E, Viarouge C, Dominguez M, Hendrikx P, Calavas D, Desprat A, Languille J, Comtet L, Pourquier P, Eleouët JF, Delmas B, Marianneau P, Vitour D, Zientara S (2013). Epidemiology, molecular virology and diagnostics of Schmallenberg virus. Vet Res.

[16] Bilk S, Schulze C, Fischer M, Beer M, Hlinak A, Hoffmann B (2012). Organ distribution of Schmallenberg virus RNA in malformed newborns. Vet Microbiol.

[17] Bréard E, Lara E, Comtet L, Viarouge C, Doceul V, Desprat A, Vitour D, Pozzi N, Cay AB, De Regge N, Pourquier P, Schirrmeier H, Hoffmann B, Beer M, Sailleau C, Zientara S: **Validation of a Commercially Available Indirect Elisa Using a Nucleocapside Recombinant Protein for Detection of Schmallenberg Virus Antibodies.** *PLoS ONE* 2013, **8**(1). doi:10.1371/journal.pone.0053446. 10.1371/journal.pone.0053446 PMC3546048 23335964

[18] Dominguez M, Calavas D, Jaÿ M, Languille J, Fediaevsky A, Zientara S, Hendrikx P, Touratier A (2012). Preliminary estimate of Schmallenberg virus infection impact in sheep flocks. France Vet Rec.

[19] OIE (2012). OIE scientists review knowledge on Schmallenberg virus [Internet].

[20] Zanella G, Raballand C, Durand B, Sailleau C, Pelzer S, Benoit F, Doceul V, Zientara S, Bréard E: **Likely introduction date of Schmallenberg virus into France according to monthly serological surveys in cattle.** *Transbound Emerg Dis* 2013. doi:10.1111/tbed.12198. 10.1111/tbed.12198 24330549

[21] Gache K, Dominguez M, Pelletier C, Petit E, Calavas D, Hendrikx P, Touratier A (2013). Schmallenberg virus: a seroprevalence survey in cattle and sheep, France, winter 2011–2012. Vet Rec.

[22] GDS France, Plateforme ESA (2014). Surveillance de l’infection congénitale par le virus Schmallenberg – Saison III - Bilan au 4 février 2014.

[23] Martinelle L, Dal Pozzo F, Gauthier B, Kirscvink N, Saegerman C: **Field veterinary survey on clinical and economic impact of Schmallenberg virus in Belgium.** *Transbound Emerg Dis* 2012. doi:10.1111/tbed.12030. 10.1111/tbed.12030 23279714

[24] Lievaart-Peterson K, Luttikholt S, Van den Brom R, Vellema P (2012). Schmallenberg virus infection in small ruminants, first review of the situation and prospects in Northern Europe. Small Rum Res.

[25] Saegerman C, Martinelle L, Dal Pozzo F, Kirschvink N: **Preliminary survey on the impact of Schmallenberg virus on sheep flocks in south of Belgium.** *Transbound Emerg* 2013. Dis. doi:10.1111/tbed.12047. 10.1111/tbed.12047 23294537

